# G4STAB: a multi-input deep learning model to predict G-quadruplex thermodynamic stability based on sequence and salt concentration

**DOI:** 10.1093/bioinformatics/btaf545

**Published:** 2025-09-27

**Authors:** Donn Liew, Akesha Dinuli Dharmatilleke, Edwin See, Ee Hou Yong

**Affiliations:** Division of Physics and Applied Physics, School of Physical and Mathematical Sciences, Nanyang Technological University, Singapore 637371, Singapore; Division of Physics and Applied Physics, School of Physical and Mathematical Sciences, Nanyang Technological University, Singapore 637371, Singapore; Division of Physics and Applied Physics, School of Physical and Mathematical Sciences, Nanyang Technological University, Singapore 637371, Singapore; Division of Physics and Applied Physics, School of Physical and Mathematical Sciences, Nanyang Technological University, Singapore 637371, Singapore

## Abstract

**Motivation:**

G-quadruplexes (G4s) are non-canonical nucleic acid structures formed in guanine-rich regions that modulate gene regulation and genomic stability. The thermodynamic stability of G4s directly influences their biological functions and potential as therapeutic targets. However, current quantitative frameworks for predicting G4 stability rely on predetermined structural features, limiting their effectiveness for diverse G4 topologies, and fail to account for environmental factors such as ion concentration and pH that significantly modulate G4 stability in cellular contexts.

**Results:**

We present G4STAB, a multi-input deep learning neural network that accurately predicts DNA G4 melting temperatures based on sequence features, salt concentration, and pH. Trained on 2382 diverse DNA G4 sequences, our model achieves high accuracy (*R*${\;}^{2}=0.8$) without relying on predetermined G4 structural features. G4STAB successfully captures established G4 stability determinants and proposes previously unobserved sequence–stability relationships. Analysis of 391 502 experimentally validated G4s reveals that cancer-like ionic environments alter G4 stability profiles, with a 13.5-fold increase in the number of structures exhibiting physiological melting temperatures (36–42°C). These findings suggest systematic genomic patterns in G4 stability responses across chromosomes and gene types.

**Availability and implementation:**

G4STAB is available at https://github.com/donn-liew/G4STAB; G4STAB web database interface is available at https://donn-liew.github.io/g4stab-web-database/.

## 1 Introduction

G-quadruplexes (G4s) are non-canonical nucleic acid structures with characteristic secondary (Popenda *et al.* 2020) and tertiary features (Lu 2020, Zok *et al.* 2022) that serve as crucial regulatory elements in biological systems. These structures form in guanine-rich sequences through assembly of four guanine bases into planar G-tetrads, which stack via π−π interactions and are stabilized by monovalent cations such as K${\;}^{+}$ and Na${\;}^{+}$ (Ying *et al.* 2003). Originally identified in telomeric regions (Sundquist and Klug 1989, Williamson *et al.* 1989, Schaffitzel *et al.* 2001), G4s occur throughout the genome, including oncogene promoters (Eddy and Maizels 2006) and immunoglobulin switch regions (Moccia *et al.* 2019). Their prevalence in regulatory regions indicates fundamental roles in transcriptional regulation (Siddiqui-Jain *et al.* 2002, Huppert and Balasubramanian 2007, Thakur *et al.* 2009, Petr *et al.* 2016, Mao *et al.* 2018), telomere maintenance (Eversole and Maizels 2000, Jansson *et al.* 2019), DNA replication (Paeschke *et al.* 2011, Brosh Jr 2013), and genomic instability (Eddy and Maizels 2006, De Magis *et al.* 2019, Wang *et al.* 2019, Rider *et al.* 2022).

G4s have emerged as potential therapeutic targets due to their biological relevance (Eversole and Maizels 2000, Sun *et al.* 2008, Wang *et al.* 2019). Understanding factors that influence G4 stability is crucial for rational drug design and intervention strategies (Risitano and Fox 2003, Bugaut and Balasubramanian 2008, Stegle *et al.* 2009). G4 stability is influenced by multiple factors operating at both molecular and cellular levels. At the molecular level, G4 structural stability is governed by primary sequence determinants such as the number of consecutive guanines forming G-quartets (Rachwal *et al.* 2007a, Pandey *et al.* 2013), the nucleotide length of loop regions connecting these G-quartets (Olsen *et al.* 2006), and the nucleotide composition within these loop regions (Pandey *et al.* 2013). Environmental conditions such as ionic environment, pH (Yan *et al.* 2013, Galer *et al.* 2016, 2023, Benabou *et al.* 2019) and molecular crowding agents (Arora and Maiti 2009) also modulate G4 stability. Monovalent cations K${\;}^{+}$ and Na${\;}^{+}$ are crucial for G4 stability because they coordinate with the central cavity of G-quartets to stabilize the structure, with K${\;}^{+}$ providing greater stabilization compared to Na${\;}^{+}$ (Guédin *et al.* 2008, Smargiasso *et al.* 2008, Wang *et al.* 2011, Ma *et al.* 2019, Moccia *et al.* 2019, Luo *et al.* 2024).

Cancer cells exhibit distinctive ionic environments that may significantly impact G4 stability. Reduced Na${\;}^{+}$/K${\;}^{+}$–ATPase expression across malignancies (Nagy *et al.* 1981, Zs.-Nagy *et al.* 1983, Suhail 2010) disrupts ionic homeostasis, increasing intracellular Na${\;}^{+}$/K${\;}^{+}$ ratios. Since K${\;}^{+}$ stabilizes G4 structures more effectively than Na${\;}^{+}$, these altered conditions may affect G4 stability. Normal cells maintain K${\;}^{+}$ at 140–150 mM and Na${\;}^{+}$ at 10–15 mM (Jansson 1990), but cancer cells show consistent ionic shifts: thyroid cancer (K${\;}^{+}$: 95–105 mM, Na${\;}^{+}$: 30–45 mM) (Zs.-Nagy *et al.* 1983), urogenital cancers (K${\;}^{+}$: 95–102 mM, Na${\;}^{+}$: 30–45 mM) (Nagy *et al.* 1981), and breast cancer (K${\;}^{+}$: 80–100 mM, Na${\;}^{+}$: 23–34.5 mM) (Cameron *et al.* 1980, Ianniello *et al.* 2021). These elevated Na${\;}^{+}$/K${\;}^{+}$ ratios across cancer types suggest that understanding G4 stability modulation in cancerous ionic environments could reveal therapeutic vulnerabilities (Mijatovic *et al.* 2008).

Although the qualitative effects of these factors on G4 stability are well documented, existing quantitative frameworks have significant limitations. Previous methods, including Gaussian process regression (GP) (Stegle *et al.* 2009) and G4Boost (Cagirici *et al.* 2022), achieved progress in G4 stability prediction but share several constraints. Both rely on predefined canonical features (G-tetrad stacks, loop lengths) and restrictive motif patterns, making them less effective for non-canonical G4 structures increasingly identified in genomic contexts (Chambers *et al.* 2015, Piazza *et al.* 2017). Furthermore, these approaches are unable to account for environmental factors such as ion concentrations and pH levels that significantly influence G4 stability.

We address these limitations by developing G4STAB (G4 STABility predictor), a multi-input deep learning neural network trained on 2382 DNA G4 sequences that integrates sequence characteristics with experimental parameters including salt concentration and pH. G4STAB accurately predicts melting temperatures ($T_{m}$) across varying ionic conditions for both canonical and non-canonical DNA G4 variants without relying on predetermined structural features. This tool predicts DNA G4 stability under varying ionic conditions to support G4 research.

## 2 Materials and methods

### 2.1 Compilation of G-quadruplex dataset

**Figure 1.**
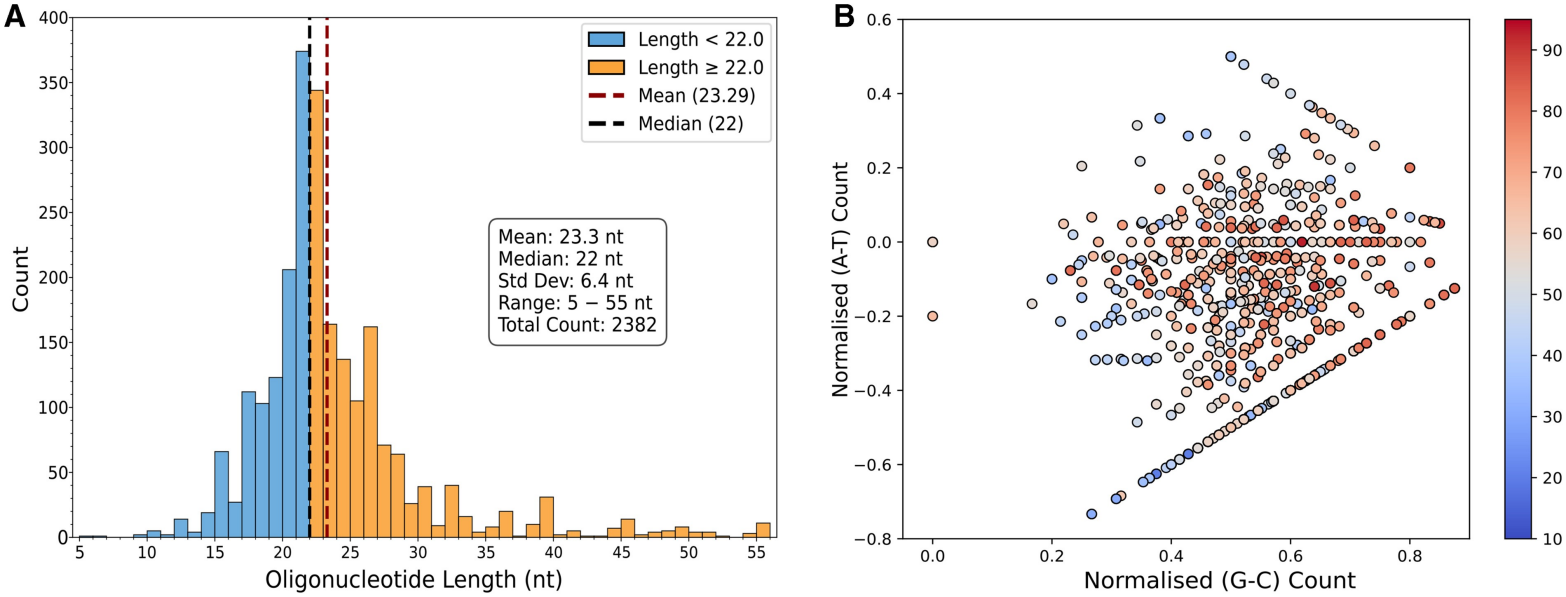
Characteristics of the G4 dataset used in this study and its sequence composition analysis. (A) Sequence length distribution of 2382 G4 sequences showing a prominent peak at 20–24 nucleotides (mean: 23.4 nt, median: 22 nt, standard deviation: 7.6 nt), where length refers to the number of nucleotides in each sequence. (B) Scatter plot showing normalized nucleotide content differences: G minus C content difference (G−C)/length versus A minus T content difference (A−T)/length. Points are coloured by melting temperatures (10–90°C), revealing distinct clustering patterns and correlation between nucleotide content and thermal stability.

We conducted an extensive review of existing literature (published before December 2024) on G4 stability, collating 2382 data points from published studies to form the G4 dataset (Fig. 1). Each data point consists of four key experimental parameters: G4 DNA primary sequence, melting temperature $T_{m}$, pH level and buffer (cation) concentration. $T_{m}$ data were compiled from studies that conducted various experimental techniques, including UV melting (Ying *et al.* 2003, Guédin *et al.* 2008, Luo *et al.* 2024), CD melting (Lopes-Nunes *et al.* 2020), DSC (Pagano *et al.* 2013), and FRET melting (Rachwal *et al.* 2007b, Wang *et al.* 2011). Although different experimental methods were used to obtain these data points, these experimental methods measured $T_{m}$ (i.e. temperature at which 50% of the quadruplex has unfolded). Due to the limited number of experimental measurements using Li${\;}^{+}$ or NH4${\;}^{+}$, these two cation types were grouped into a single category to ensure sufficient data for meaningful analysis (You *et al.* 2017). Entries with duplicates in primary sequence, salt concentration and pH levels were omitted from the dataset to prevent biased weighting during model training and potential overestimation of performance in the k−fold cross-validation analysis. Studies involving molecular crowding agents such as ethylene glycol or polyethylene glycol (PEG) were excluded from the dataset, as these co-solutes can significantly alter G4 stability through osmotic pressure effects and dehydration. The full G4 dataset used in this study can be found in Table 1, available as supplementary data at *Bioinformatics* online.

### 2.2 Feature extraction and encoding

G4 sequences were standardized to 100 nucleotides using symmetric padding and processed using one-hot encoding (4×100 matrix per sequence) and k−mer encoding with lengths 1–4, yielding 340 k−mer features (4 monomers, 16 dimers, 64 trimers, 256 tetramers) compiled into a 20×17 matrix. Salt concentrations were encoded as 1×3 vectors representing K${\;}^{+}$, Na${\;}^{+}$, and Li${\;}^{+}$/NH${\;}_{4}^{+}$ concentrations extracted from experimental conditions. All concentration and pH values were normalized using min-max scaling to [0, 1] range.

### 2.3 G4STAB neural network architecture

**Figure 2.**
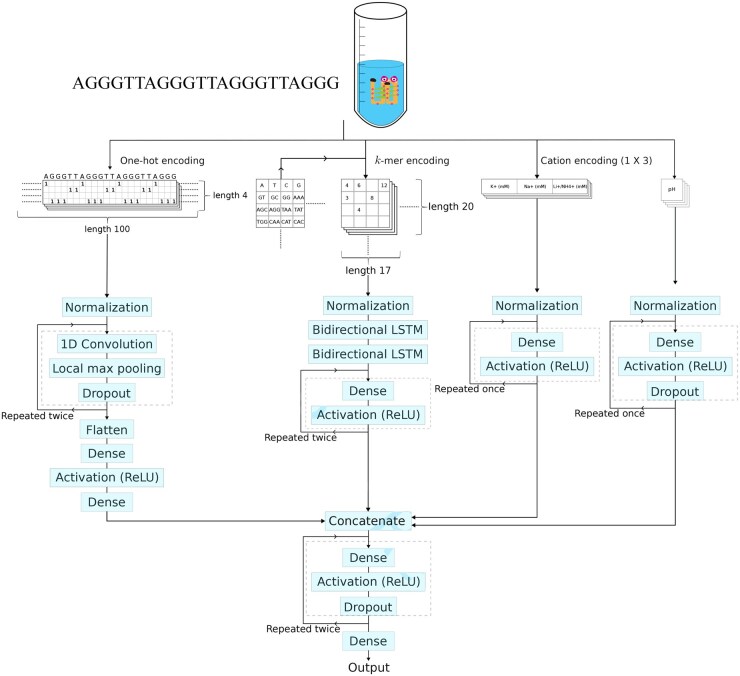
G4STAB neural network architecture and processing workflow. The architecture comprises four parallel input branches processing different feature types, illustrated using the putative G4 sequence AG_3_T_2_AG_3_T_2_AG_3_T_2_AG_3_ as an example. *First branch* (leftmost) processes one-hot encoded sequences of length 100 through a series of transformations. Input sequences undergo normalization before passing through a 1D convolution layer, followed by local max pooling and dropout. This process is repeated. Finally, the resulting features are flattened and passed through two dense layers with a ReLU activation function between them. *Second branch*: processes *k*-mer features via normalization before passing through two bidirectional LSTM layers. The output is processed by three dense layers with ReLU activation. *Third branch*: processes salt concentration data through a simple feed-forward network. The input data is normalized and passed through two dense layers, each followed by ReLU activation functions. *Fourth branch*: handles pH values through a feed-forward network with dropout regularization. After normalization, the data passes through two dense layers with ReLU activations, each followed by a dropout layer. *Final processing*: outputs from all four branches are concatenated and fed through a series of fully connected layers. This combined representation is processed through two dense layers with ReLU activation, followed by a dropout layer for regularization. The final output layer consists of a single dense unit with linear activation to predict $T_{m}$.

G4STAB was implemented using Python and the TensorFlow Keras framework (Fig. 2). Training was performed on an NVIDIA A100 Tensor Core GPU (SXM4) with 40GB HBM2e memory. The Nadam optimizer (Adam with Nesterov momentum) was configured with a learning rate $\eta =1.55\times 10^{-4}$, momentum decay rates $\beta_{1}=0.995$, $\beta_{2}=0.9915$ and weight decay $\lambda =4\times 10^{-5}$. The small learning rate (η = 1.55 × 10${\;}^{-4}$) prevents gradient conflicts between sequence and environmental features, while the high $\beta_{1}$ (0.995) provides strong momentum for navigating the multi-input G4 stability prediction. These hyperparameters were determined through systematic Bayesian optimization across an extensive parameter space.

### 2.4 Training and evaluation of G4STAB

G4STAB was evaluated using 5-fold cross-validation and repeated 200 times. The test fold was further randomly split, with 50% of the test fold retained for final testing and the remainder 50% used as a validation set to monitor model performance during training. For each run, the model was reinitialized and trained with the Nadam optimizer with a mean squared error (MSE) loss function. Performance was measured using root mean squared error (RMSE) and coefficient of determination (R^2^) averaged across all 200 runs.

### 2.5 Genomic-scale stability prediction using trained G4STAB models

G4STAB was used to predict the stability of experimentally validated G4 (eG4) sequences using an ensemble-based approach to maximize prediction reliability. The ensemble consists of 100 independently trained G4STAB models, each initialized with identical architecture but different random weights. Each model was then trained on random subsets of the G4 dataset (similar to Section 2.4).

Each eG4 sequence was processed by all 100 models simultaneously to predict $T_{m}$. The final $T_{m}$ for each sequence was calculated as the arithmetic mean of all 100 individual model predictions. This ensemble-based stability prediction approach was applied to analyse G4 stability principles (Section 3.3), large-scale prediction of G4 stability across the human genome (Section 3.4) and analysis of endogenous G4s in cancer-like ionic environments (Section 3.5).

### 2.6 Analysis of G4 structures across multiple ionic conditions in the human genome

We utilized eG4 sequences from the human genome through the EndoQuad database (Qian *et al.* 2024). Our analysis focused on the Human eG4.txt dataset which contained 391 502 entries. Each entry contained genomic positions, stability measurements and identification numbers. To assess G4 stability across physiologically relevant conditions, we applied G4STAB under four ionic conditions. (i) high potassium condition or normal condition (100 mM K${\;}^{+}$, 0 mM Na${\;}^{+}$, 0 mM Li${\;}^{+}$/NH4${\;}^{+}$) representing the standard potassium-rich environment used for in vitro G4 studies and approximates intracellular conditions (Ying *et al.* 2003, Sun *et al.* 2008, Tran *et al.* 2021); (ii) moderate potassium condition (50 mM K${\;}^{+}$, 0 mM Na${\;}^{+}$, 0 mM Li${\;}^{+}$/NH4${\;}^{+}$) representing sufficient K${\;}^{+}$ for most G4s to fold (Brown *et al.* 2005, Hatzakis *et al.* 2010, Noer *et al.* 2016, You *et al.* 2017); (iii) sodium-only condition (0 mM K${\;}^{+}$, 100 mM Na${\;}^{+}$, 0 mM Li${\;}^{+}$/NH4${\;}^{+}$) for studying G4 behaviour without potassium; and (iv) cancer-like ionic conditions (70 mM K${\;}^{+}$, 30 mM Na${\;}^{+}$, 0 mM Li${\;}^{+}$) modelling the altered Na${\;}^{+}$/K${\;}^{+}$ ratio (0.43) observed across multiple cancer types (Cameron *et al.* 1980, Nagy *et al.* 1981, Zs.-Nagy *et al.* 1983, Suhail 2010, Ianniello *et al.* 2021) (see Section 3.4 for rationale).

We focused our comparative analysis on normal and cancer-like ionic conditions. The $T_{m}$ distributions of 391 502 G4s were analysed under both conditions using histograms with 1°C bins to visualize the complete temperature spectrum. Further analyses examined the chromosomal and gene-type distributions of G4s with physiological $T_{m}$ (36–42°C). For gene-type classification, categories were ordered according to total G4 abundance.

## 3 Results and discussion

### 3.1 G-quadruplex dataset characteristics

Our dataset of 2382 G4 sequences comprises both intramolecular and intermolecular structures. Combining data from multiple experimental techniques expands the dataset and enables stability analysis. Although experimental variations might introduce method-specific biases, $T_{m}$ values do not deviate significantly when different techniques are applied to identical G4 structures under similar buffer conditions (Olsen *et al.* 2009, Gray and Chaires 2011, Tucker *et al.* 2018, Moccia *et al.* 2019).

The sequence length distribution (Fig. 1A) is slightly right-skewed, with the highest frequency between 22–25 nucleotides (mean: 23.4 nt, median: 22.0 nt, SD: 7.6 nt, IQR: 5 nt). While most sequences cluster within the 22–25 nt range, smaller secondary peaks around 40–55 nt likely represent more complex G4 structures. This distribution is heavily skewed towards shorter oligonucleotides (mean: 23.4 nt), potentially limiting generalizability to longer G4-forming sequences. Shorter sequences predominantly represent simpler G4 topologies, while longer genomic G4s may adopt more complex conformations, including multiple domains or hybrid structures underrepresented in our training data. This bias likely reduces prediction accuracy for sequences exceeding 50 nucleotides, which constitute only 1% (24 out of 2382 sequences) of our dataset. The bias reflects experimental limitations in G4 research, where longer sequences present synthesis costs and measurement difficulties. Genomic G4s are embedded within longer sequence contexts where flanking regions may influence stability, unlike isolated short oligonucleotides. Future model iterations would benefit from expanded training data incorporating longer G4-forming sequences to enhance genomic application accuracy.

$T_{m}$ distribution spans 84°C $\left(\max(T_{m})-\min(T_{m})\right)$ with average $T_{m}$ of 56.5°C and a standard deviation (SD) of 13.2°C, highlighting the diverse range of G4 structures in our dataset. Notably, G4STAB does not require DNA concentration as input due to the established biophysical principle that $T_{m}$ of unimolecular G4 structures remain concentration-independent (Mergny *et al.* 1998, Hatzakis *et al.* 2010), unlike duplex DNA transitions (Privalov and Crane-Robinson 2018). While our dataset includes sequences shorter than 21 nucleotides (28.8%) that might form intermolecular structures, shorter sequences were retained to maintain a sizeable G4 dataset.

A scatter plot (Fig. 1B) was used to further characterize the sequence properties in the dataset. We plot normalized nucleotide count differences for each sequence, where normalization was performed by dividing by the total sequence length. The x−axis represents the normalized difference between G and C content ((G minus C)/length), ranging from 0 to 0.8, while the y−axis shows the normalized A and T difference ((A minus T)/length), ranging from −0.8 to 0.6. Each point represents a single sequence, with its $T_{m}$ indicated by colour (10–90°C, blue to red). The visualization reveals that sequences cluster in three linear arrangements: sequences with low A-T ratios (A minus T ≈−0.6), sequences with balanced A-T content (A minus T ≈ 0), and sequences with moderate A-T excess (0.2 < A minus T < 0.4). Sequences with higher thermal stability (>70°C, orange-red points) concentrate in the high G-C difference region (0.4–0.8), reflecting that guanine-rich sequences are more thermally stable.

### 3.2 Performance evaluation demonstrates G4STAB’s predictive capabilities and constraints

G4STAB (Fig. 2) achieves strong predictive performance with RMSE of 5.96°C, MAE of 4.42°C, and *R*^2^ of 0.8, with 70% of predictions falling within ±5°C of experimental values. Additional cross-validation results for k=2 to k=4 are provided in Fig. 1, available as supplementary data at *Bioinformatics* online.

**Figure 3.**
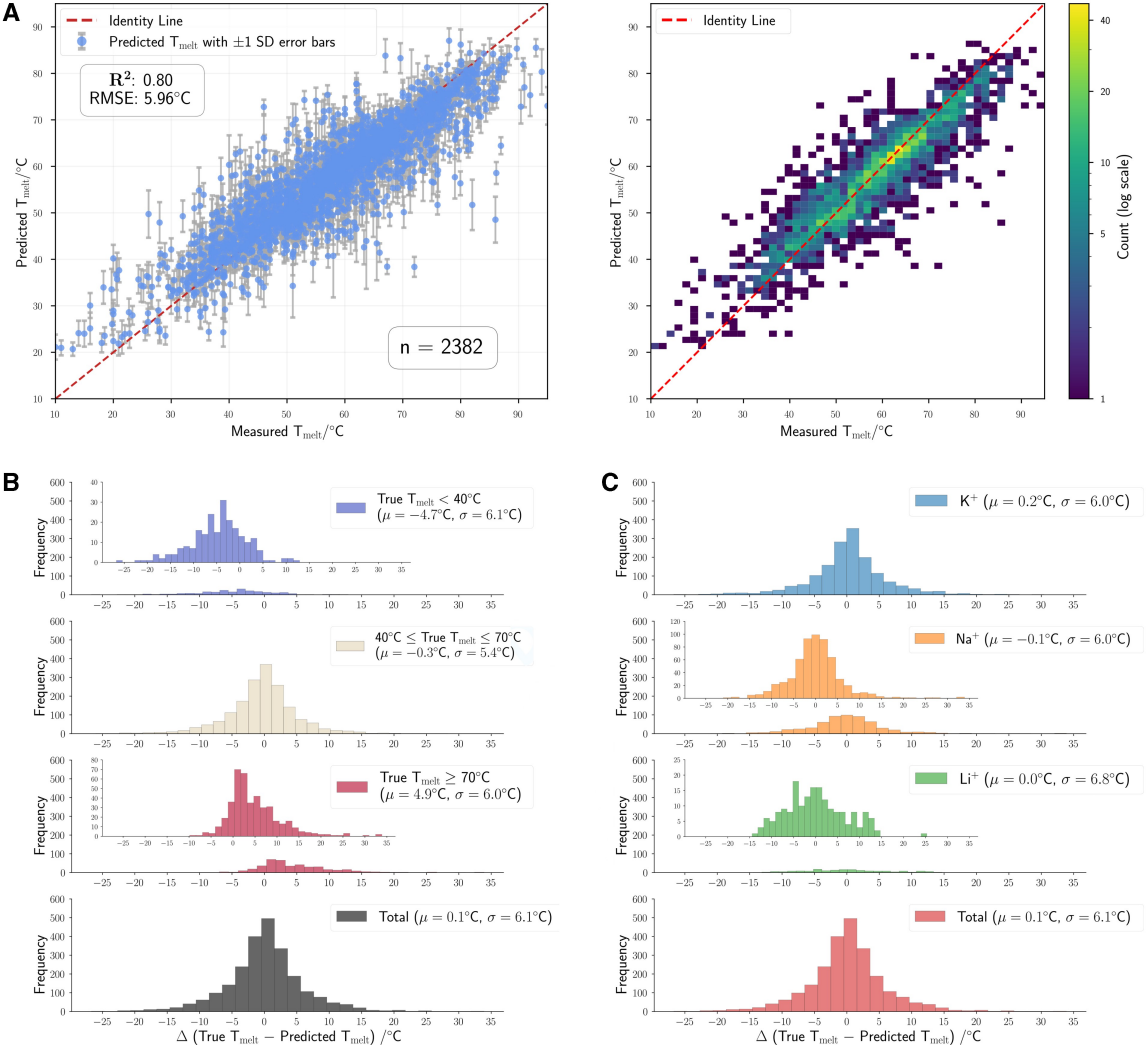
G4STAB demonstrates high predictive accuracy for G4 $T_{m}$. (A) True (experimental) vs predicted $T_{m}$ with ±1 SD error bars; the dotted diagonal is the identity line (y=x). (B) Distribution of prediction errors, $\Delta T=T_{\text{true}}-T_{\text{pred}}$, stratified by $T_{\text{true}}$ ranges: $T_{\text{true}}<40^{{}^{\circ}}C$, $40^{{}^{\circ}}C\leq T_{\text{true}}\leq 70^{{}^{\circ}}C$, $T_{\text{true}}>70^{{}^{\circ}}C$, and the overall distribution. (C) Distribution of ΔT across monovalent cations (K${\;}^{+}$, Na${\;}^{+}$, Li${\;}^{+}$), and the overall distribution.

G4STAB shows optimal performance in the 40–70°C range (Fig. 3A), with prediction uncertainty at temperature extremes due to limited training samples (n=226, 9.5% below 40°C; n=409, 17.2% above 70°C). G4s with $T_{m}$ below 40°C are systematically overpredicted (left-skewed errors) while high $T_{m}$ G4s (>70°C) tend towards underprediction (right-skewed errors) (Fig. 3B). These systematic biases suggest future model refinements should focus on improving prediction accuracy at temperature extremes.

G4STAB maintains consistent performance across ionic conditions (Fig. 3C), with similar error distributions for $K^{+}$ (mean = −0.2, σ = 6.4), Na${\;}^{+}$ (mean = 0.1, σ = 6.2), and NH_4_/Li${\;}^{+}$ (mean = 0.3, σ = 6.5) solutions. This robust performance, regardless of monovalent cation identity, is particularly noteworthy given known differential cation effects on G4 stability.

**Figure 4.**
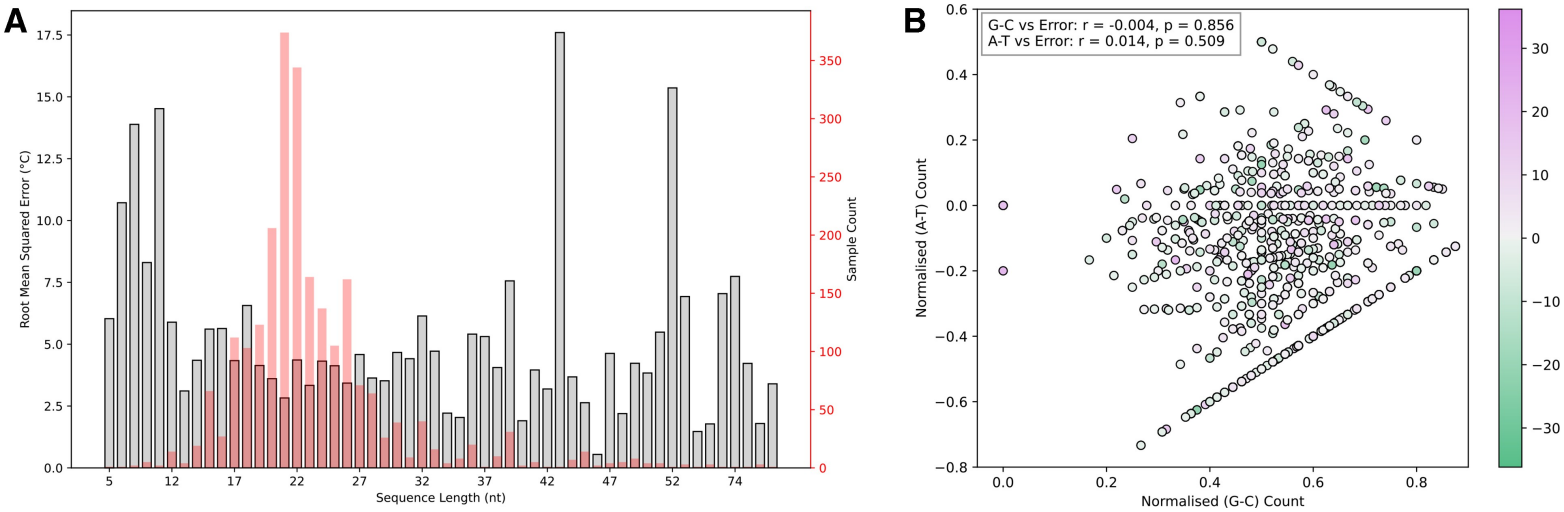
G4 sequence length and nucleotide composition effects on $T_{m}$ prediction error. (A) RMSE between predicted and experimental $T_{m}$ (gray bars, primary y−axis) and sample distribution (red bars, secondary y−axis) across G4 DNA sequences of lengths (5–99 nucleotides). Data represents 2382 G4 DNA sequences with 500 independent predictions averaged per sequence. (B) Scatter plot of normalized G–C (G minus C) count vs normalized A–T (A minus T) count for 2382 G4 sequences. Points are coloured by $T_{m}$ prediction error (experimental $T_{m}$ minus predicted $T_{m}$) using a diverging colour scale from negative (green) to positive (purple) errors. Correlation coefficients: G–C vs Error: Pearson r=−0.004, P=0.856; A–T vs Error: r=−0.014, P=0.509.

G4STAB effectively captures sequence-structure-stability relationships across diverse contexts. Despite dataset dominance by sequences 21 nucleotides long (374 sequences), RMSE remains consistent across 5–99 nucleotides (Fig. 4A). Nucleotide composition analysis (Fig. 4B) reveals no systematic bias, with no significant correlations between G–C content and prediction errors (Pearson r=−0.004, P=0.856) or A–T content and errors (r=0.014, P=0.509). We did not evaluate the correlation between prediction accuracy and G-tract length because our dataset lacks structural characterization data. Without NMR or X-ray crystallography, it would be difficult to determine which G-tracts participate in folding versus remain as loops, given that 69.9% of sequences in our dataset contain non-canonical features. Similarly, we did not evaluate the correlation between prediction accuracy and different loop types (bulges, lateral, diagonal, propeller loops) since such structural characterization is unavailable in our dataset. While databases like ONQUADRO (Zok *et al.* 2022) (615 entries) and DSSR-G4DB (Lu 2020) (556 entries) contain structural information, they are limited and lack systematic thermodynamic data, making accurate correlation analysis challenging.

Determining individual feature importance within G4STAB proved challenging due to the interdependence between sequence-based features. When a sequence is modified, both one-hot encoding and k−mer representations transform simultaneously according to deterministic rules, preventing isolation of each feature type’s independent contribution. Additionally, deep learning models operate as “black boxes” where standard feature importance methods cannot effectively isolate specific feature contributions (Ewald *et al.* 2024). Nevertheless, G4STAB successfully learned several stability principles extending beyond established structure-stability relationships, as demonstrated in subsequent analyses.

While pH levels were included as a feature in G4STAB, their overall contribution to prediction accuracy appears relatively minor compared to sequence features and cation concentrations. This lower importance likely stems from the limited pH diversity in our dataset. Although the data spans the full biological range (4.0–8.0), 89% of samples were measured at narrow pH conditions (7.0–7.5), with particularly strong clustering at pH 7.0 (28%), 7.2 (37%), and 7.4 (23%). Several potentially relevant pH values (5.0, 5.5, and 6.5) are entirely absent from the dataset. This distribution bias does not discount the role of pH in modulating G4 structure and stability (Yan *et al.* 2013, Galer *et al.* 2016, 2023, Benabou *et al.* 2019) but rather reflects experimental practices in G4 research, limiting our ability to accurately assess pH’s contribution to G4 stability prediction. Future studies focusing specifically on pH-responsive G4 structures could better interpret these relationships.

### 3.3 Analysis of G-quadruplex stability principles with G4STAB

**Figure 5.**
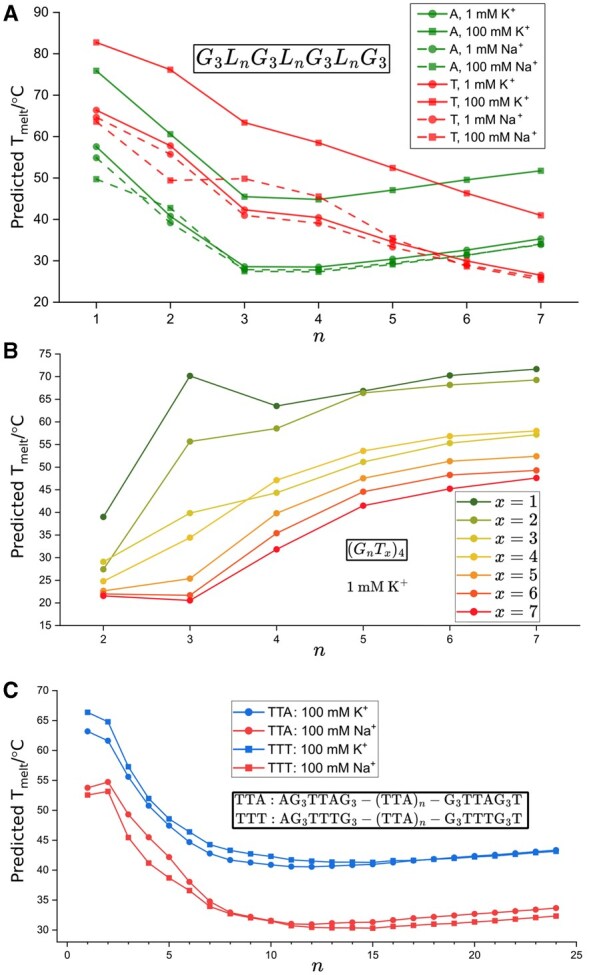
G4STAB’s prediction reflects observed G4 stability patterns in experimentation. (A) Thermal stability of G4s sequence motifs G_3_L${\;}_{n}$G_3_L${\;}_{n}$G_3_L${\;}_{n}$G_3_, where loop lengths L${\;}_{n}$ vary from n∈{1,2,…,7} nucleotides. Results are shown for adenine loops (green) and thymine loops (red) with varying K${\;}^{+}$/Na${\;}^{+}$ concentrations. (B) Stability analysis of G4s with repeating (G${\;}_{n}$T${\;}_{x}$)_4_ motifs, where G-tract length n∈{2,3…,7} (x−axis) and thymine stretches x∈{1,2…,7}. (C): Effect of the central loop ${\left(\text{TTA}\right)}_{n}$ on G-quadruplex stability in structures with sequence patterns ${\text{AG}}_{3}{\text{TTAG}}_{3}{\left(\text{TTA}\right)}_{n}G_{3}{\text{TTAG}}_{3}T$ and ${\text{AG}}_{3}{\text{TTTG}}_{3}{\left(\text{TTA}\right)}_{n}G_{3}{\text{TTTG}}_{3}T$, where n∈{1,3…,25}, under different ionic conditions.

Figure 5A illustrates the relationship between loop length and G4 stability with sequence pattern G_3_L${\;}_{n}$G_3_L${\;}_{n}$G_3_L${\;}_{n}$G_3_, where loop lengths L${\;}_{n}$ vary from n∈{1,2,…,7}. G4STAB captured the inverse relationship between loop length and thermal stability in intramolecular G4s, with single-nucleotide extensions decreasing $T_{m}$ by 11–18°C before reaching a plateau, comparable to experimental $T_{m}$ decreases of 8–16°C (Bugaut and Balasubramanian 2008). G4STAB reproduced this inverse correlation across different loop compositions and cation conditions, correctly predicting A loops’ destabilizing effect compared to T loops at shorter lengths (n=1–5). Temperature differences were 3–19°C, consistent with experimental $T_{m}$ decreases of 6–8°C (Rachwal *et al.* 2007b, Guédin *et al.* 2008). K${\;}^{+}$ consistently provided greater stabilization than Na${\;}^{+}$ across all loop lengths (approximately 1.5°C at 1 mM, 17–18°C at 100 mM concentration), matching experimental observations (Guédin *et al.* 2008, Smargiasso *et al.* 2008, Moccia *et al.* 2019).

New patterns emerged from G4STAB analysis. G4s with all-A loops show stability minima at loop length n=4, after which stability increases, while G4s with all-T loops show consistent destabilization with increasing loop length to n=7. T-only loops exhibit greater structural sensitivity to length variations (40–50°C range) compared to A-only loops (23–33°C range), suggesting T loops confer greater structural flexibility. While higher K${\;}^{+}$ concentration consistently increases stability by 15–16°C, Na${\;}^{+}$ concentration shows variable effects and may decrease stability, particularly for G4s with A loops at n=1 (−6.75°C) and T loops at n=2 (−7.44°C). The interaction between base type and cation type varies across loop lengths, with the largest interaction effects observed at n=2 (−4.23°C) and n=7 (+2.19°C).

Figure 5B examines G4s with repeating ${\left(G_{n}T_{x}\right)}_{4}$ motifs, where G-tract length n∈{2,3,…,7} and thymine stretches T∈{1,2,…,7}. G4STAB successfully predicts the non-monotonic relationship between G-tract length and stability for sequences with a single thymine loop (x=1), where three consecutive guanines provide optimal thermal stability compared to both shorter and longer G-tracts. This finding aligns with experimental stability measurements that have identified (G_3_T)_4_ as “anomalously stable” with the order of stability n=3>n=7>n=6>n=5>n=4 (Risitano and Fox 2003, Rachwal *et al.* 2007a, Pandey *et al.* 2013). For sequences with TT loop (x=2), stability increases monotonically with G-tract length, while for longer thymine loops, the relationship becomes non-linear. G4STAB demonstrates that while increasing G-tract length may enhance stability, this effect plateaus with longer G-tracts, likely due to increased conformational entropy costs that counterbalance the favourable stacking interactions (Hazel *et al.* 2004). Conversely, increasing T repeats generally reduces stability, with this destabilizing effect most pronounced in structures with 3 G-tracts (n=3) that experience a 70.7% decrease in stability from x=1 to x=7. While 3 G-tracts provide optimal stability efficiency (stability per guanine) for sequences with a single T loop, 3 G-tracts destabilize rapidly with increasing T loop length, falling below 50% of maximum at x=4, a pattern not observed with other G-tract lengths. Longer G-tracts (n≥5) maintain consistent stability across varying thymine loop lengths, suggesting they provide structural resilience against loop length variations.

Figure 5C reveals the critical role of central loop length in determining overall G4 stability. The model predicts stability patterns for structures with sequence patterns ${\text{AG}}_{3}{\text{TTAG}}_{3}{\left(\text{TTA}\right)}_{n}G_{3}{\text{TTAG}}_{3}T$ and ${\text{AG}}_{3}{\text{TTTG}}_{3}{\left(\text{TTA}\right)}_{n}G_{3}\text{TT}$

${\text{TG}}_{3}T$ , where n∈{1,3…,25}. The results demonstrate an inverse relationship between central loop length and thermal stability, with longer central loops progressively destabilizing the G4 structure. Furthermore, G4STAB correctly predicts the persistence of cation-specific effects (K${\;}^{+}$ vs Na${\;}^{+}$) across the entire range of central loop lengths, with K${\;}^{+}$ consistently providing greater stabilization regardless of loop composition or length (Hao *et al.* 2019). The destabilizing effect of central loop length is more pronounced in TTT-containing structures than TTA structures.

Figure 5C shows that the relationship between central loop length and stability is non-linear. The rate of destabilization is higher for shorter central loops (n=1 to n=10, with −2.6°C to −2.9°C decrease per *n* increase) compared to longer loops (n=10 to n=24), where stability initially plateaus and increases slightly (0.1°C to 0.2°C per *n* increase). Both TTA and TTT G4 structures exhibit distinct minimum stability points (at n=12 and n=15, respectively) after which stability modestly increases with additional loop length. At n=17, TTA structures in K${\;}^{+}$ become more stable than TTT structure, inverting the stability relationship observed at shorter loop lengths. Additionally, TTT G4 structures show greater cation sensitivity (average K${\;}^{+}$/Na${\;}^{+}$ difference of 11.0°C for TTT vs 8.8°C for TTA structures).

These findings demonstrate that G4STAB captures established stability determinants while revealing previously uncharacterized sequence-dependent patterns. G4STAB successfully predicts G4 stability across diverse structural variations, potentially complementing experimental approaches in understanding G4 stability.

### 3.4 Large-scale genomic analysis reveals chromosome-specific and gene-type-specific G4 stability patterns

G4STAB was applied to 391 502 eG4s sequences to analyse stability patterns across the human genome under normal and cancer-like ionic conditions described in Section 2.6. In particular, cancer-like ionic conditions represent a simplified model of altered ionic homeostasis observed across diverse cancer types. While exact ionic concentrations vary considerably between cancer types, stages, and tumour microenvironments, the proposed cancer-like ionic environment has a Na${\;}^{+}$/K${\;}^{+}$ ratio of 0.43, which falls within the range observed in thyroid cancer (0.29–0.47) (Zs.-Nagy *et al.* 1983), urogenital cancers (0.29–0.47) (Nagy *et al.* 1981), and breast cancer (0.23–0.43) (Cameron *et al.* 1980, Ianniello *et al.* 2021). The 30% reduction in K${\;}^{+}$ from normal conditions reflects cancer-associated decreases in Na${\;}^{+}$/K${\;}^{+}$-ATPase function (Nagy *et al.* 1981, Zs.-Nagy *et al.* 1983, Suhail 2010), while the Na${\;}^{+}$ concentration of 30 mM falls within the range reported across multiple cancer types (23–45 mM) (Cameron *et al.* 1980, Nagy *et al.* 1981, Zs.-Nagy *et al.* 1983, Ianniello *et al.* 2021). The proposed cancer-like ionic condition represents a simplified model, as ionic environments vary considerably between different cancer types and within individual tumours. This heterogeneity could result in variable G4 stability profiles across tumour regions, potentially leading to different levels of G4-dependent gene expression within the same tumour.

**Figure 6.**
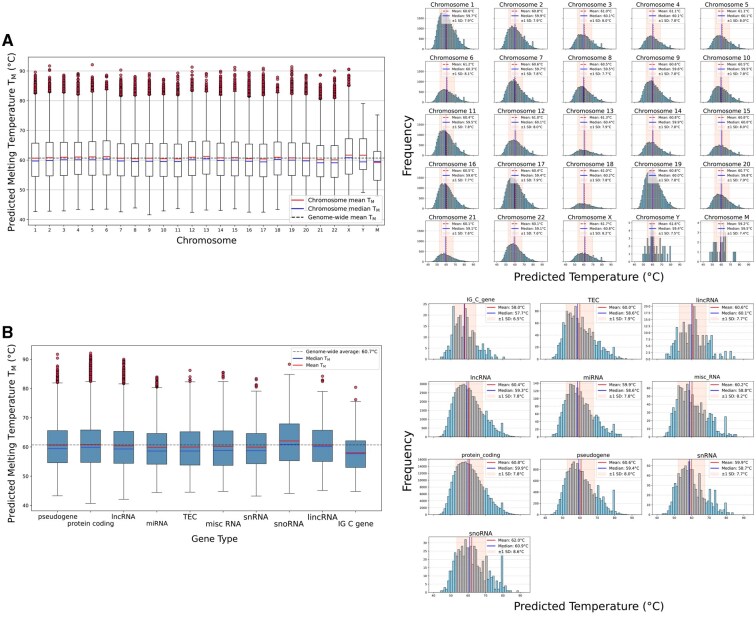
Distribution of predicted G4 $T_{m}$ across human chromosomes and gene types. (A) Left: boxplots of predicted G4 $T_{m}$ for each chromosome at 100 mM K${\;}^{+}$, with outliers shown as filled circles. Right: histograms displaying frequency distribution with dashed lines (mean), solid lines (median) and light shading (±1 SD). (B) Left: boxplots of predicted $T_{m}$ across gene types (minimum 100 G4 occurrences, from 391 502 total G4 structures), with outliers as filled circles. Right: histograms for each gene type with red lines (mean), blue lines (median), and light shading (±1 SD).

Analysis of G4 $T_{m}$ across human chromosomes reveals distinct stability patterns (Fig. 6A). Across the entire human genome, average $T_{m}$ was 60.7°C at 100 mM K${\;}^{+}$. Sex chromosomes X and Y displayed the highest mean temperatures at 61.7°C, while the mitochondrial chromosome (M) showed the lowest at 59.2°C, suggesting potential evolutionary and functional significance. Among autosomes, chromosomes 13 and 6 exhibited the highest mean temperatures (61.3°C and 61.2°C respectively), while chromosomes 22 and 21 had the lowest (60.1°C and 60.2°C, respectively).

Analysis across gene types with a minimum of 100 G4 occurrences (Fig. 6B) revealed that snoRNAs contain G4s with the highest mean $T_{m}$ (62.0°C) and broadest distribution (SD =8.6°C), while immunoglobulin constant genes (IG C) contain G4s with the lowest mean $T_{m}$ (58.0°C) and narrowest distribution (SD =6.5°C). Protein-coding genes and pseudogenes showed intermediate mean $T_{m}$ of 60.8°C and 60.6°C, respectively. Different classes of non-coding RNAs demonstrated varying stability profiles, with lincRNAs maintaining a higher average $T_{m}$ (60.6°C) compared to miRNAs and snRNAs (both at 59.9°C). Kruskal–Wallis tests confirmed significant differences in G4 Tm distributions across gene types (H=258.41, $P=1.7\times 10^{-50}$), though effect sizes were small $\left(\eta^{2}=0.0006\right)$. The 4.0°C difference between the highest (snoRNAs) and lowest (IG C genes) mean $T_{m}$ values may reflect functional adaptation to their respective cellular environments and regulatory roles.

### 3.5 Altered ionic environments in cancer cells significantly impact G4 stability profiles

**Figure 7.**
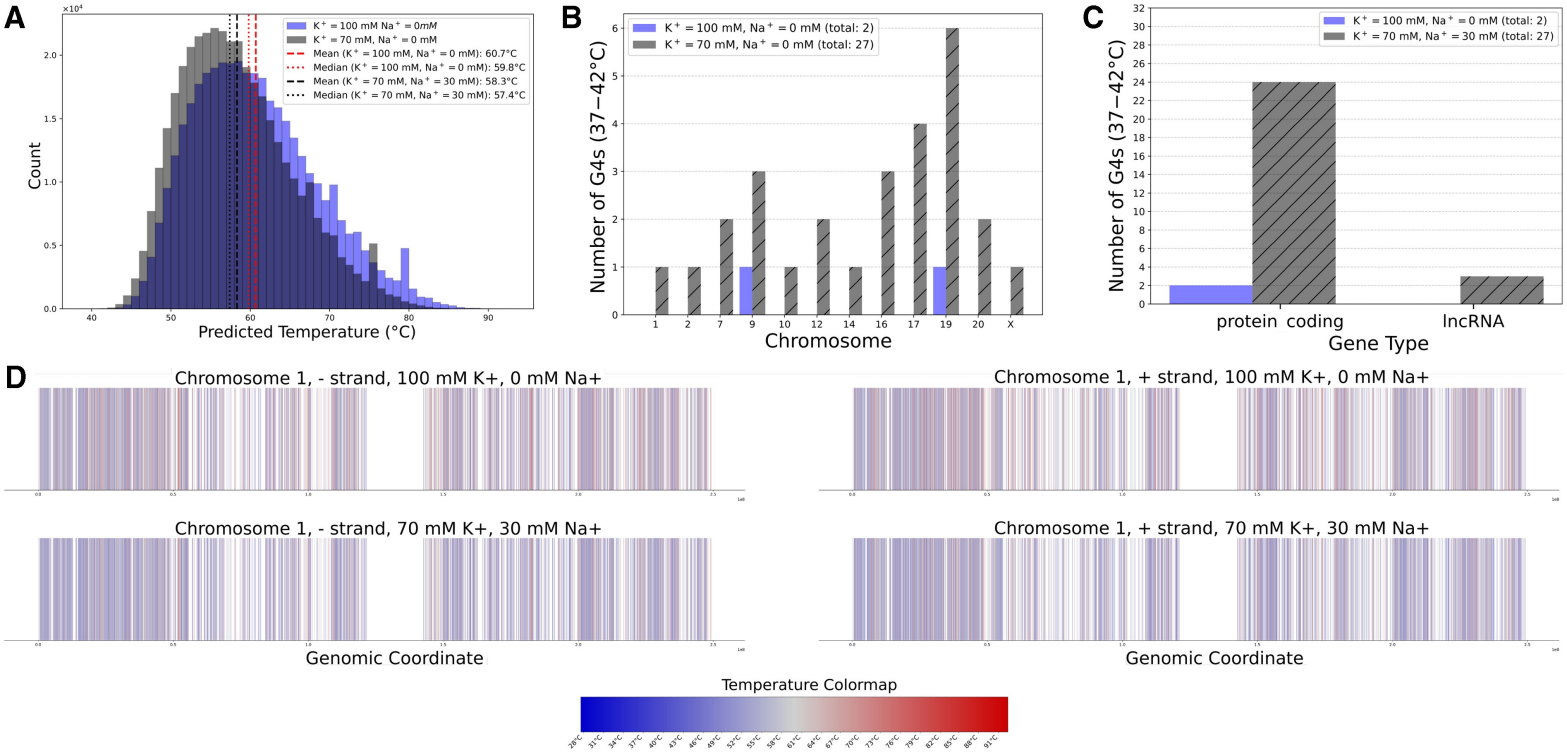
Cancer-like ionic environment significantly impacts G4 stability profiles. (A) Histogram of predicted $T_{m}$ for 391 502 endogenous G4 sequences under normal (100 mM K${\;}^{+}$, blue) versus cancer-like ionic conditions (70 mM K${\;}^{+}$, 30 mM Na${\;}^{+}$, black). (B) Chromosomal distribution of endogenous G4s with physiological melting temperatures (36–42°C). Bar chart comparing G4 counts across human chromosomes under normal cellular conditions (100 mM K${\;}^{+}$) versus cancer-like conditions (70 mM K${\;}^{+}$, 30 mM Na${\;}^{+}$, hatched). (C) Distribution of G4s with physiological $T_{m}$ (36–42°C) across gene types under normal cellular and cancer-like ionic conditions. Bar chart comparing G4 counts across gene types under normal cellular conditions (100 mM K${\;}^{+}$) versus cancer-like conditions (70 mM K${\;}^{+}$, 30 mM Na${\;}^{+}$, hatched). (D) Chromosome ideograms comparing G4 positional distribution and predicted $T_{m}$ on chromosome 1 under different ionic conditions. Top row: normal cellular conditions (100 mM K${\;}^{+}$); bottom row: cancer-like conditions (70 mM K${\;}^{+}$, 30 mM Na${\;}^{+}$). Left: negative strand; right: positive strand. The ideogram shows concentration patterns across chromosome 1, with position 228–229 megabases (Mb) containing 1089 G4s. For genome-wide ideograms, please refer to Figs 2 and 3, available as supplementary data at Bioinformatics online.

Comparing normal with cancer-like ionic conditions, we observed a leftward shift in the melting temperature distribution for 391 502 eG4 sequences (Fig. 7A). $T_{m}$ predictions exhibited slight positive skewness under normal cellular conditions (skewness = 0.52) and cancer-like conditions (skewness = 0.58). Shapiro-Wilk tests confirmed non-normal distribution for both conditions (W=0.9765, $P=6.1\times 10^{-28}$ for normal; W=0.9749, $P=8.1\times 10^{-29}$). $T_{m}$ decreased from 60.7°C/59.8°C (mean/median) to 58.3°C/57.4°C, an average decrease of 2.4°C. SD narrowed from 7.9°C to 7.1°C, suggesting more uniform destabilization. Levene’s test confirmed significant variance differences between conditions (W=3780.84, P<0.0001). Welch’s *t*-test verified statistical significance (t=139.25, df=782570, P<0.0001), with 95% confidence interval (CI) for mean difference of 2.3°C to 2.4°C and moderate effect size (Cohen’s d=0.31).

We observe a 13.5-fold increase in G4s exhibiting physiological $T_{m}$ (36–42°C), from 2 under normal cellular conditions to 27 under cancer-like conditions (Fig. 7B). While this (an increase from 2 to 27) represents a small absolute number of sequences, it may suggest a potential mechanism by which altered ionic homeostasis under cancer-like conditions could modulate G4-dependent gene regulation. This suggests a potential mechanism by which altered ionic homeostasis under cancer-like conditions could modulate G4-dependent gene regulation. The increase suggests that G4 structures stable under normal cellular conditions may become dynamically regulated under cancer-like ionic conditions, potentially affecting G4-mediated processes.

Chromosomal distribution analysis reveals non-random patterns with biological significance. Under cancer-like conditions, chromosomes 19, 17, and 16 harboured the highest numbers of physiological $T_{m}$ G4s (6, 4, and 3 sequences, respectively). Chromosome 19, despite its relatively small size, showed the largest absolute increase (+5 G4s), suggesting concentrated regions of G4 sensitivity to ionic changes. This increase is noteworthy given chromosome 19’s gene-dense nature and enrichment for cancer-associated genes (Varis *et al.* 2003, Grimwood *et al.* 2004, Wang *et al.* 2015, Jinesh *et al.* 2018, Cao *et al.* 2020). The concentration of physiologically-melting G4s in protein-coding genes (24 of 27), rather than being distributed randomly across genomic regions, suggests non-random effects concentrated in protein-coding genes.

Gene-type distribution analysis demonstrated that protein-coding genes contained the majority of physiologically-melting G4s under cancer-like conditions (24 of 27), followed by lncRNAs (3 of 27) (Fig. 7C). Under normal ionic conditions, only protein-coding genes contained G4s with physiological $T_{m}$. This differential distribution suggests that G4 stability in regulatory non-coding regions may be particularly sensitive to ionic changes typical of cancer environments. Fisher’s exact test showed no statistically significant difference between conditions (P=1.0), but the complete absence of lncRNA G4s in this temperature range under normal conditions compared to their presence under cancer-like conditions (3/27 total) suggests potential shifts in G4 stability profiles. Detailed analysis across gene types revealed statistically significant differences in G4 stability within both normal conditions (Kruskal-Wallis test: H=258.41, P<0.0001) and cancer-like conditions (H=261.32, P<0.0001), though with modest effect sizes ($\eta^{2}=0.0006$ for both). Statistically significant differences in gene type responses to cancer-like ionic conditions were observed (Kruskal-Wallis test on $T_{m}$ shifts: H=198.10, P<0.0001, $\eta^{2}=0.0005$). While all gene types showed significant destabilization in cancer-like conditions (all P<0.0001), magnitude varied, with snoRNAs showing the largest mean $T_{m}$ decrease (2.5°C, 95% CI: 1.5°C to 3.4°C) and IG C genes showing the smallest (2.2°C, 95% CI: 1.1°C to 3.2°C). IG C genes maintained the lowest mean Tm in both conditions (58.0°C normal, 55.8°C cancer-like) while snoRNAs exhibited the highest (62.0°C normal, 59.6°C cancer-like). Most gene types showed significantly different variances between conditions (Levene’s test, P<0.05), with only lincRNA (W=1.96, P=0.16) and IG C genes (W=2.95, P=0.09) maintaining homogeneous variances, suggesting differential sensitivity to ionic changes across gene categories.

Examination of chromosome 1 (Fig. 7D; genome-wide ideograms in Figs 2 and 3, available as supplementary data at *Bioinformatics* online) revealed position-specific variations in ionic sensitivity. Telomeric regions (1–3 megabases) containing ∼6000 G4s showed consistent 2.3–2.5°C decreases in $T_{m}$ under cancer-like conditions. Other regions displayed varying responses, with positions 175–176 Mb showing 1.9°C decreases, while positions 244–245 Mb maintained high stability (61.8°C, about 3.5°C above chromosome-wide average). These position-specific variations suggest differential ionic sensitivity across chromosomal regions.

### 3.6 A web resource of G4 melting temperatures predicted by G4STAB

**Figure 8.**
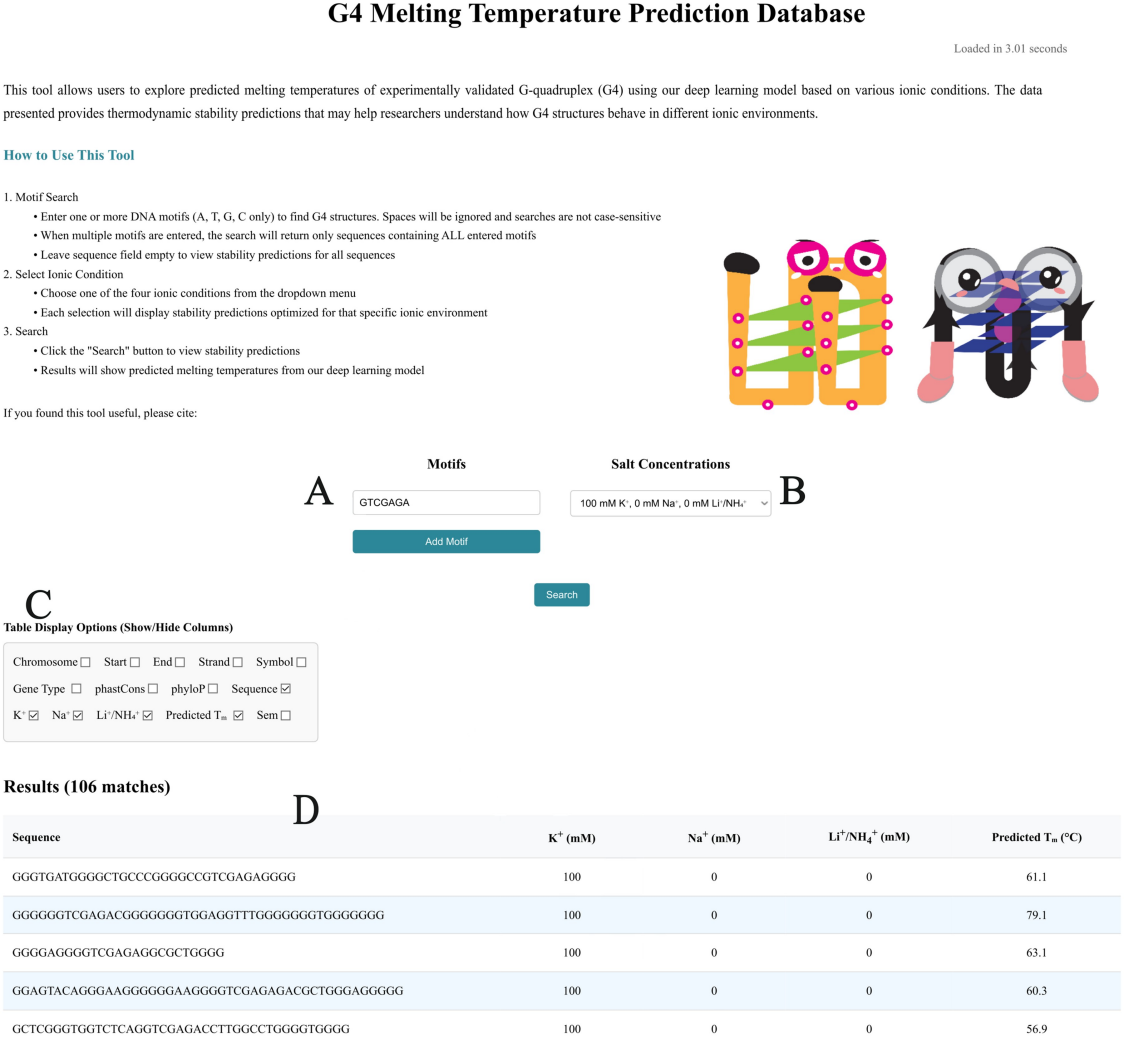
Interactive web tool for G-quadruplex (G4) $T_{m}$ prediction (available at: https://donn-liew.github.io/g4stab-web-database/). This interface allows users to search for G4 sequences and predict their melting temperatures under various ionic conditions. The tool features a motif search functionality (A) where multiple motifs can be specified, with matched sequences required to contain all specified subsequences. Users can select from four different ionic environments with varying concentrations of K${\;}^{+}$, Na${\;}^{+}$, and Li${\;}^{+}$/NH${\;}_{4}^{+}$ via a dropdown menu (B). The interface provides column visibility controls (C), allowing users to customize the data display by showing or hiding specific data fields such as chromosome location, gene information, conservation scores, and ionic conditions. Search results display predicted $T_{m}$ generated by G4STAB (D), enabling researchers to compare G4 stability across different sequences and salt conditions.

We developed a web-based database interface (available at: https://donn-liew.github.io/g4stab-web-database/) that provides researchers with access to $T_{m}$ predictions from G4STAB for 391 502 experimentally validated G4 sequences under four ionic conditions. The database includes genomic annotations such as chromosome location, strand information, gene association, and conservation scores (phastCons and phyloP), with each prediction accompanied by standard error of measurement (SEM). The interface (Fig. 8) enables users to search for specific DNA motifs within G4 sequences and filter results based on ionic conditions. Results are displayed in an interactive table with customizable column visibility, allowing researchers to focus on genomic context, sequence composition, or thermodynamic properties according to their research needs.

## 4 Conclusion

G4STAB represents a significant advancement in predicting G4 stability through its multi-input deep learning architecture that integrates sequence features with environmental parameters, including salt concentration and pH. Using an expanded dataset of 2382 G4 sequences, G4STAB achieves high predictive performance (*R*${\;}^{2}=$0.8) across diverse sequence contexts and experimental conditions without relying on predetermined structural features.

G4STAB successfully captures established principles governing G4 stability while revealing previously unobserved patterns, including non-linear relationships between central loop length and thermal stability as well as sequence context effects beyond canonical G-tracts. G4STAB also demonstrates differential impacts of cation type across G-tetrad number loop composition and loop length variations.

Our large-scale genomic analysis of 391 502 eG4s provides new insights into stability patterns across the human genome, revealing chromosome-specific and gene type-specific variations in G4 thermal profiles. These findings may advance our understanding of how G4 structures that affect gene regulation and genomic stability are distributed across different genomic contexts.

Our findings demonstrate that cancer-like ionic environments alter G4 stability profiles, resulting in a 13.5-fold increase in G4s with physiological $T_{m}$ (36–42°C), though this is based on a limited number of sequences. This suggests that altered ionic homeostasis under cancer-like conditions could modulate G4-dependent processes.

The G4STAB web interface provides researchers with easy access to pre-computed G4 stability predictions for specific sequences and genomic analyses. G4STAB advances our understanding of G4 biology and may inform future therapeutic strategies targeting these important regulatory structures.

## Supplementary Material

btaf545_Supplementary_Data

## Data Availability

G4STAB is available at https://github.com/donn-liew/G4STAB; G4STAB web database interface is available at https://donn-liew.github.io/g4stab-web-database/.

## References

[btaf545-B1] Arora A , Maiti S. Stability and molecular recognition of quadruplexes with different loop length in the absence and presence of molecular crowding agents. J Phys Chem B 2009;113:8784–92. 19480441 10.1021/jp809486g

[btaf545-B2] Benabou S , Mazzini S, Aviñó A et al A pH-dependent bolt involving cytosine bases located in the lateral loops of antiparallel g-quadruplex structures within the smarca4 gene promotor. Sci Rep 2019;9:15807. 31676783 10.1038/s41598-019-52311-5 PMC6825181

[btaf545-B3] Brosh RM Jr . DNA helicases involved in DNA repair and their roles in cancer. Nat Rev Cancer 2013;13:542–58. 23842644 10.1038/nrc3560 PMC4538698

[btaf545-B4] Brown NM , Rachwal PA, Brown T et al Exceptionally slow kinetics of the intramolecular quadruplex formed by the oxytricha telomeric repeat. Org Biomol Chem 2005;3:4153–7. 16267597 10.1039/b511706b

[btaf545-B5] Bugaut A , Balasubramanian S. A sequence-independent study of the influence of short loop lengths on the stability and topology of intramolecular DNA G-quadruplexes. Biochemistry 2008;47:689–97. 18092816 10.1021/bi701873c PMC2408741

[btaf545-B6] Cagirici HB , Budak H, Sen TZ. G4boost: a machine learning-based tool for quadruplex identification and stability prediction. BMC Bioinformatics 2022;23:240. 35717172 10.1186/s12859-022-04782-z PMC9206279

[btaf545-B7] Cameron IL , Smith NKR, Pool TB et al Intracellular concentration of sodium and other elements as related to mitogenesis and oncogenesis in vivo. Cancer Res 1980;40:1493–500. 7370987

[btaf545-B8] Cao C , Hong P, Huang X et al HPV-ccdc106 integration alters local chromosome architecture and hijacks an enhancer by three-dimensional genome structure remodeling in cervical cancer. J Genet Genomics 2020;47:437–50. 33023834 10.1016/j.jgg.2020.05.006

[btaf545-B9] Chambers VS , Marsico G, Boutell JM et al High-throughput sequencing of DNA G-quadruplex structures in the human genome. Nat Biotechnol 2015;33:877–81. 26192317 10.1038/nbt.3295

[btaf545-B10] De Magis A , Manzo SG, Russo M et al DNA damage and genome instability by g-quadruplex ligands are mediated by R loops in human cancer cells. Proc Natl Acad Sci USA 2019;116:816–25. 30591567 10.1073/pnas.1810409116 PMC6338839

[btaf545-B11] Eddy J , Maizels N. Gene function correlates with potential for G4 DNA formation in the human genome. Nucleic Acids Res 2006;34:3887–96. 16914419 10.1093/nar/gkl529 PMC1557811

[btaf545-B12] Eversole A , Maizels N. In vitro properties of the conserved mammalian protein hnRNP D suggest a role in telomere maintenance. Mol Cell Biol 2000;20:5425–32. 10891483 10.1128/mcb.20.15.5425-5432.2000 PMC85994

[btaf545-B13] Ewald FK , Bothmann L, Wright MN et al A guide to feature importance methods for scientific inference. In: World Conference on Explainable Artificial Intelligence, Valletta, Malta, July 17-19, 2024. Cham: Springer Nature Switzerland, 2024, 440–64.

[btaf545-B14] Galer P , Wang B, Šket P et al Reversible pH switch of two-quartet g-quadruplexes formed by human telomere. Angew Chem 2016;128:2033–7. 10.1002/anie.201507569 26836334

[btaf545-B15] Galer P , Wang B, Plavec J et al Unveiling the structural mechanism of a G-quadruplex pH–driven switch. Biochimie 2023;214:73–82. 37573019 10.1016/j.biochi.2023.08.002

[btaf545-B16] Gray RD , Chaires JB. Analysis of multidimensional G-quadruplex melting curves. Curr Protoc Nucleic Acid Chem 2011;17–4. 10.1002/0471142700.nc1704s45 PMC3398426 21638271

[btaf545-B17] Grimwood J , Gordon LA, Olsen A et al The DNA sequence and biology of human chromosome 19. Nature 2004;428:529–35. 15057824 10.1038/nature02399

[btaf545-B18] Guédin A , De Cian A, Gros J et al Sequence effects in single-base loops for quadruplexes. Biochimie 2008;90:686–96. 18294461 10.1016/j.biochi.2008.01.009

[btaf545-B19] Hao F , Ma Y, Guan Y. Effects of central loop length and metal ions on the thermal stability of G-quadruplexes. Molecules 2019;24:1863. 31096553 10.3390/molecules24101863 PMC6571788

[btaf545-B20] Hatzakis E , Okamoto K, Yang D. Thermodynamic stability and folding kinetics of the major g-quadruplex and its loop isomers formed in the nuclease hypersensitive element in the human c-myc promoter: effect of loops and flanking segments on the stability of parallel-stranded intramolecular g-quadruplexes. Biochemistry 2010;49:9152–60. 20849082 10.1021/bi100946g PMC2964402

[btaf545-B21] Hazel P , Huppert J, Balasubramanian S et al Loop-length-dependent folding of G-quadruplexes. J Am Chem Soc 2004;126:16405–15. 15600342 10.1021/ja045154j

[btaf545-B22] Huppert JL , Balasubramanian S. G-quadruplexes in promoters throughout the human genome. Nucleic Acids Res 2007;35:406–13. 17169996 10.1093/nar/gkl1057 PMC1802602

[btaf545-B23] Ianniello C , Moy L, Fogarty J et al Multinuclear MRI to disentangle intracellular sodium concentration and extracellular volume fraction in breast cancer. Sci Rep 2021;11:5156. 33664340 10.1038/s41598-021-84616-9 PMC7933187

[btaf545-B24] Jansson B. Dietary, total body, and intracellular potassium-to-sodium ratios and their influence on cancer. Cancer Detect Prev 1990;14:563–5. 2224920

[btaf545-B25] Jansson LI , Hentschel J, Parks JW et al Telomere DNA G-quadruplex folding within actively extending human telomerase. Proc Natl Acad Sci USA 2019;116:9350–9. 31019071 10.1073/pnas.1814777116 PMC6510993

[btaf545-B26] Jinesh GG , Flores ER, Brohl AS. Chromosome 19 miRNA cluster and CEBPB expression specifically mark and potentially drive triple negative breast cancers. PLoS One 2018;13:e0206008. 30335837 10.1371/journal.pone.0206008 PMC6193703

[btaf545-B27] Lopes-Nunes J , Carvalho J, Figueiredo J et al Phthalocyanines for G-quadruplex aptamers binding. Bioorg Chem 2020;100:103920. 32413624 10.1016/j.bioorg.2020.103920

[btaf545-B28] Lu X-J. Dssr-enabled innovative schematics of 3D nucleic acid structures with pymol. Nucleic Acids Res 2020;48:e74. 32442277 10.1093/nar/gkaa426 PMC7367123

[btaf545-B29] Luo Y , Živković ML, Wang J et al A sodium/potassium switch for G4-prone g/c-rich sequences. Nucleic Acids Res 2024;52:448–61. 37986223 10.1093/nar/gkad1073 PMC10783510

[btaf545-B30] Ma G , Yu Z, Zhou W et al Investigation of Na+ and K+ competitively binding with a G-quadruplex and discovery of a stable k+–na+-quadruplex. J Phys Chem B 2019;123:5405–11. 31244096 10.1021/acs.jpcb.9b02823

[btaf545-B31] Mao S-Q , Ghanbarian AT, Spiegel J et al DNA G-quadruplex structures mold the DNA methylome. Nat Struct Mol Biol 2018;25:951–7. 30275516 10.1038/s41594-018-0131-8 PMC6173298

[btaf545-B32] Mergny J-L , Phan A-T, Lacroix L. Following g-quartet formation by UV-spectroscopy. FEBS Lett 1998;435:74–8. 9755862 10.1016/s0014-5793(98)01043-6

[btaf545-B33] Mijatovic T , Ingrassia L, Facchini V et al Na+/k+-atpase α subunits as new targets in anticancer therapy. Expert Opin Ther Targets 2008;12:1403–17. 18851696 10.1517/14728222.12.11.1403

[btaf545-B34] Moccia F , Platella C, Musumeci D et al The role of g-quadruplex structures of ligs-generated aptamers R1. 2 and r1. 3 in IgM-specific recognition. Int J Biol Macromol 2019;133:839–49. 31022491 10.1016/j.ijbiomac.2019.04.141 PMC6548653

[btaf545-B35] Nagy IZ , Lustyik G, Nagy VZ et al Intracellular Na+: K+ ratios in human cancer cells as revealed by energy dispersive X-ray microanalysis. J Cell Biol 1981;90:769–77. 7287822 10.1083/jcb.90.3.769 PMC2111914

[btaf545-B36] Noer SL , Preus S, Gudnason D et al Folding dynamics and conformational heterogeneity of human telomeric g-quadruplex structures in Na+ solutions by single molecule fret microscopy. Nucleic Acids Res 2016;44:464–71. 26615192 10.1093/nar/gkv1320 PMC4705662

[btaf545-B37] Olsen CM , Gmeiner WH, Marky LA. Unfolding of g-quadruplexes: energetic, and ion and water contributions of g-quartet stacking. J Phys Chem B 2006;110:6962–9. 16571009 10.1021/jp0574697

[btaf545-B38] Olsen CM , Lee H-T, Marky LA. Unfolding thermodynamics of intramolecular G-quadruplexes: base sequence contributions of the loops. J Phys Chem B 2009;113:2587–95. 19014184 10.1021/jp806853n

[btaf545-B39] Paeschke K , Capra JA, Zakian VA. DNA replication through G-quadruplex motifs is promoted by the *Saccharomyces cerevisiae* PIF1 DNA helicase. Cell 2011;145:678–91. 21620135 10.1016/j.cell.2011.04.015 PMC3129610

[btaf545-B40] Pagano B , Randazzo A, Fotticchia I et al Differential scanning calorimetry to investigate G-quadruplexes structural stability. Methods 2013;64:43–51. 23500655 10.1016/j.ymeth.2013.02.018

[btaf545-B41] Pandey S , Agarwala P, Maiti S. Effect of loops and g-quartets on the stability of RNA g-quadruplexes. J Phys Chem B 2013;117:6896–905. 23683360 10.1021/jp401739m

[btaf545-B42] Petr M , Helma R, Polášková A et al Wild-type p53 binds to MYC promoter G-quadruplex. Biosci Rep 2016;36:e00397. 27634752 10.1042/BSR20160232 PMC5064454

[btaf545-B43] Piazza A , Cui X, Adrian M, Samazan F, Heddi B, Phan A-T, Nicolas AG. Non-canonical G-quadruplexes cause the hCEB1 minisatellite instability in *Saccharomyces cerevisiae*. Elife 2017;6:e26884. 28661396 10.7554/eLife.26884 PMC5491262

[btaf545-B44] Popenda M , Miskiewicz J, Sarzynska J et al Topology-based classification of tetrads and quadruplex structures. Bioinformatics 2020;36:1129–34. 31588513 10.1093/bioinformatics/btz738 PMC7031778

[btaf545-B45] Privalov PL , Crane-Robinson C. Translational entropy and DNA duplex stability. Biophys J 2018;114:15–20. 29320682 10.1016/j.bpj.2017.11.003 PMC5773757

[btaf545-B46] Qian SH , Shi M-W, Xiong Y-L et al Endoquad: a comprehensive genome-wide experimentally validated endogenous G-quadruplex database. Nucleic Acids Res 2024;52:D72–80. 37904589 10.1093/nar/gkad966 PMC10767823

[btaf545-B47] Rachwal PA , Brown T, Fox KR. Effect of g-tract length on the topology and stability of intramolecular DNA quadruplexes. Biochemistry 2007a;46:3036–44. 17311417 10.1021/bi062118j

[btaf545-B48] Rachwal PA , Brown T, Fox KR. Sequence effects of single base loops in intramolecular quadruplex DNA. FEBS Lett 2007b;581:1657–60. 17399710 10.1016/j.febslet.2007.03.040

[btaf545-B49] Rider SD , Gadgil RY, Hitch DC et al Stable G-quadruplex DNA structures promote replication-dependent genome instability. J Biol Chem 2022;298:101947. 35447109 10.1016/j.jbc.2022.101947 PMC9142560

[btaf545-B50] Risitano A , Fox KR. Stability of intramolecular DNA quadruplexes: comparison with DNA duplexes. Biochemistry 2003;42:6507–13. 12767234 10.1021/bi026997v

[btaf545-B51] Schaffitzel C , Berger I, Postberg J et al In vitro generated antibodies specific for telomeric guanine-quadruplex DNA react with stylonychia lemnae macronuclei. Proc Natl Acad Sci USA 2001;98:8572–7. 11438689 10.1073/pnas.141229498 PMC37477

[btaf545-B52] Siddiqui-Jain A , Grand CL, Bearss DJ et al Direct evidence for a g-quadruplex in a promoter region and its targeting with a small molecule to repress c-MYC transcription. Proc Natl Acad Sci U S A 2002;99:11593–8. 12195017 10.1073/pnas.182256799 PMC129314

[btaf545-B53] Smargiasso N , Rosu F, Hsia W et al G-quadruplex DNA assemblies: loop length, cation identity, and multimer formation. J Am Chem Soc 2008;130:10208–16. 18627159 10.1021/ja801535e

[btaf545-B54] Stegle O , Payet L, Mergny J-L et al Predicting and understanding the stability of G-quadruplexes. Bioinformatics 2009;25:i374–i382. 19478012 10.1093/bioinformatics/btp210 PMC2687964

[btaf545-B55] Suhail M. Na+, k+-atpase: ubiquitous multifunctional transmembrane protein and its relevance to various pathophysiological conditions. J Clin Med Res 2010;2:1. 22457695 10.4021/jocmr2010.02.263w PMC3299169

[btaf545-B56] Sun D , Liu W-J, Guo K et al The proximal promoter region of the human vascular endothelial growth factor gene has a G-quadruplex structure that can be targeted by g-quadruplex–interactive agents. Mol Cancer Ther 2008;7:880–9. 18413801 10.1158/1535-7163.MCT-07-2119 PMC2367258

[btaf545-B57] Sundquist WI , Klug A. Telomeric DNA dimerizes by formation of guanine tetrads between hairpin loops. Nature 1989;342:825–9. 2601741 10.1038/342825a0

[btaf545-B58] Thakur RK , Kumar P, Halder K et al Metastases suppressor nm23-h2 interaction with g-quadruplex DNA within c-MYC promoter nuclease hypersensitive element induces c-myc expression. Nucleic Acids Res 2009;37:172–83. 19033359 10.1093/nar/gkn919 PMC2615625

[btaf545-B59] Tran PLT , Rieu M, Hodeib S et al Folding and persistence times of intramolecular g-quadruplexes transiently embedded in a DNA duplex. Nucleic Acids Res 2021;49:5189–201. 34009328 10.1093/nar/gkab306 PMC8136832

[btaf545-B60] Tucker BA , Hudson JS, Ding L et al Stability of the Na+ form of the human telomeric G-quadruplex: role of adenines in stabilizing G-quadruplex structure. ACS Omega 2018;3:844–55. 30023791 10.1021/acsomega.7b01649 PMC6045420

[btaf545-B61] Varis A , van Rees B, Weterman M et al DNA copy number changes in young gastric cancer patients with special reference to chromosome 19. Br J Cancer 2003;88:1914–9. 12799636 10.1038/sj.bjc.6600969 PMC2741104

[btaf545-B62] Wang L , Wen Y, Liu J et al Promoting the formation and stabilization of human telomeric G-quadruplex DNA, inhibition of telomerase and cytotoxicity by phenanthroline derivatives. Org Biomol Chem 2011;9:2648–53. 21347502 10.1039/c0ob00961j

[btaf545-B63] Wang X , Zhang Y, Nilsson CL et al Association of chromosome 19 to lung cancer genotypes and phenotypes. Cancer Metastasis Rev 2015;34:217–26. 25982285 10.1007/s10555-015-9556-2

[btaf545-B64] Wang Y , Yang J, Wild AT et al G-quadruplex DNA drives genomic instability and represents a targetable molecular abnormality in ATRX-deficient malignant glioma. Nat Commun 2019;10:943. 30808951 10.1038/s41467-019-08905-8 PMC6391399

[btaf545-B65] Williamson JR , Raghuraman MK, Cech TR. Monovalent cation-induced structure of telomeric DNA: the g-quartet model. Cell 1989;59:871–80. 2590943 10.1016/0092-8674(89)90610-7

[btaf545-B66] Yan Y-Y , Tan J-H, Lu Y-J et al G-quadruplex conformational change driven by pH variation with potential application as a nanoswitch. Biochim Biophys Acta 2013;1830:4935–42. 23811336 10.1016/j.bbagen.2013.06.019

[btaf545-B67] Ying L , Green JJ, Li H et al Studies on the structure and dynamics of the human telomeric G quadruplex by single-molecule fluorescence resonance energy transfer. Proc Natl Acad Sci USA 2003;100:14629–34. 14645716 10.1073/pnas.2433350100 PMC299749

[btaf545-B68] You J , Li H, Lu X-M et al Effects of monovalent cations on folding kinetics of G-quadruplexes. Biosci Rep 2017;37 BSR20170771/28588052 10.1042/BSR20170771 PMC5567087

[btaf545-B69] Zok T , Kraszewska N, Miskiewicz J et al Onquadro: a database of experimentally determined quadruplex structures. Nucleic Acids Res 2022;50:D253–D258. 34986600 10.1093/nar/gkab1118 PMC8728301

[btaf545-B70] Zs.-Nagy I , Lustyik G, Lukács G et al Correlation of malignancy with the intracellular Na+:K+ ratio in human thyroid tumors. Cancer Res 1983;43:5395–402. 6616471

